# Fe‐Enhanced Proton Capture on Boron Nitride Surfaces for Improved Photocatalytic Methane Conversion to C1 Chemicals

**DOI:** 10.1002/smsc.70302

**Published:** 2026-05-11

**Authors:** Yong He, Wang Yu, Yuehan Cao, Sibo Wang, Kailiang Xu, Ying Zhou

**Affiliations:** ^1^ State Key Laboratory of Oil and Gas Reservoir Geology and Exploitation Southwest Petroleum University Chengdu China; ^2^ School of New Energy and Materials Southwest Petroleum University Chengdu China

**Keywords:** methane conversion, non‐noble metal materials, photocatalysis, proton capture

## Abstract

The direct solar‐driven conversion of methane (CH_4_) into oxygenated liquid compounds has emerged as a promising approach for achieving high‐value utilization of natural gas. Here, an Fe/boron nitride (BN) catalyst system was constructed. Mechanistic investigations demonstrate that Fe acts as an acceptor of photogenerated electrons, promoting charge‐carrier separation and transfer and thereby accelerating H_2_O dissociation to generate ·OH radicals. These reactive oxygen species facilitate CH_4_ activation, leading to a notable enhancement in CH_4_ conversion. In addition, Fe sites strengthen proton capture by adjacent N atoms, promoting the formation of N—H bonds. The resulting N—H bonds can provide protons in situ, effectively suppressing the dehydrogenation of key reaction intermediates. As a result, over 1 wt% Fe/BN, the production rate of liquid oxygenates (CH_3_OH and HCHO) reaches 321.1 µmol·g^−1^·h^−1^, which is 2.3 times higher than that of pristine BN, with an overall selectivity of up to 94.2%. The synergistic interaction between Fe and BN transcends conventional electron transfer: Fe not only promotes photogenerated charge separation but, more importantly, alters the reaction pathway by modulating surface proton chemistry. This work provides a useful reference for regulating and optimizing two‐dimensional, non‐noble‐metal semiconductor materials.

## Graphical abstract

Fe has acted as an electron acceptor, leading to electron accumulation on its surface. This has enhanced proton capture by adjacent N atoms, accelerated H_2_O dissociation to form ·OH radicals, and thereby promoted CH_4_ activation. The N—H bonds formed on the boron nitride (BN) surface have supplied protons in situ, effectively suppressing overoxidation and facilitating CH_3_OH regeneration.

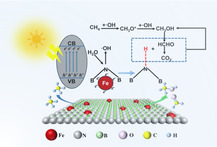

## 1 Introduction

Rapid industrialization and urbanization are driving a sustained rise in global energy demand [1]. The combustion of fossil fuels such as coal, petroleum, and natural gas has released substantial amounts of greenhouse gases into the atmosphere, triggering a series of global ecological and environmental challenges that pose serious threats to human survival and development [2, 3, 4]. Methane (CH_4_), the major constituent of natural gas and the second most influential greenhouse gas after CO_2_, is often viewed as a comparatively cleaner fossil fuel because of its low cost, high calorific value, abundant reserves, and the lowest carbon‐to‐hydrogen ratio among hydrocarbons [5]. Beyond its role as an energy carrier, CH_4_ is also a key chemical feedstock in the transition toward a renewable‐energy economy [6, 7]. Currently, ≈90% of methane worldwide is utilized through direct combustion in applications such as residential heating, daily cooking, and thermal power generation [8]. While this conventional approach can meet basic energy demands quickly, it also releases large quantities of CO_2_ during combustion [9]. Converting methane into high‐value‐added chemical products such as methanol (CH_3_OH) and formaldehyde (HCHO) could not only significantly enhance energy utilization efficiency but also reduce greenhouse gas emissions, mitigate the ecological pressure caused by the greenhouse effect [10, 11], and achieve synergistic development of economic benefits, energy security, and ecological conservation [12, 13, 14, 15].

Photocatalysis harnesses solar energy to drive chemical transformations and thus offers a sustainable route for the direct conversion of methane into value‐added chemicals under mild conditions [16, 17, 18]. By rationally designing photocatalysts that generate reactive species upon light excitation, the inert C—H bonds in CH_4_ can be activated and selectively transformed [19, 20]. In recent years, the rapid development of two‐dimensional (2D) photocatalytic materials has injected new vitality into this field [21, 22]. Their atomically thin layered structures lead to highly exposed surface atoms, providing maximized interfacial sites for reactant adsorption and catalytic reactions [23]. Simultaneously, the atomic‐scale thickness of these materials significantly shortens the migration path of photogenerated charge carriers from the bulk to the surface, effectively suppressing electron–hole recombination and contributing to improved photon utilization efficiency [24, 25, 26]. For example, Mao et al. [27] demonstrated that edge‐rich MoS_2_ with sulfur‐vacancy sites enables room‐temperature CH_4_ oxidation using O_2_, achieving >99% selectivity to C1 oxygenates while effectively suppressing deep oxidation. However, most two‐dimensional materials have relatively wide bandgaps and are responsive primarily to ultraviolet light, resulting in generally low overall methane conversion efficiency. Consequently, the strategy of “loading co‐catalytic materials” has gradually become a research focus in this field [28]. The flat and extended surface structure of two‐dimensional materials offers an ideal platform for supporting metal clusters or single atoms, which not only promotes high dispersion of the active components and prevents agglomeration but also enhances overall catalytic performance through interfacial electronic synergy [29]. Current research has largely focused on noble‐metal cocatalysts such as gold, silver, and palladium [30, 31]. While these can effectively promote methane activation, their high cost significantly limits their economic feasibility for practical applications [32, 33]. Therefore, recent studies have gradually shifted attention toward non‐noble metal systems. For instance, Ye et al. [34] reported GaN decorated with trace Co clusters for photocatalytic methane coupling, delivering stable operation over 110 h with propane selectivity up to 90.6% and negligible byproducts.

Building on the promise of two‐dimensional semiconductors for photocatalytic methane conversion, boron nitride (BN) is an attractive yet underexplored platform in this materials family [35]. Recent studies show that BN can intrinsically resist overoxidation during C1–C6 alkane conversion, maintaining unusually low CO_2_ formation, and its robust 2D framework further supports catalyst engineering [36]. Nevertheless, pristine BN still requires optimization to improve light utilization, charge separation, and the controlled generation of reactive oxygen species for efficient methane upgrading [37]. Here, we introduce earth‐abundant Fe onto BN nanosheets via photodeposition to create highly dispersed Fe sites that promote interfacial charge transfer and regulate surface proton chemistry. Consequently, the optimal 1 wt% Fe/BN delivers a total oxygenate (CH_3_OH + HCHO) formation rate of 321.1 µmol·g^−1^·h^−1^ with 94.2% selectivity, while effectively suppressing deep oxidation. Mechanistic studies indicate that Fe modulates the electronic structure of BN and acts as an electron acceptor to enhance charge separation; meanwhile, it strengthens proton capture at adjacent N sites to accelerate H_2_O activation and enrich ·OH‐related reactive species for C—H activation, with surface N—H species supplying in situ protons to protect key intermediates from overoxidation. This work highlights “proton management” on a nonoxide semiconductor as a design principle to simultaneously boost activity and preserve oxygenate selectivity in solar‐driven methane conversion.

## 2 Results and Discussion

### 2.1 Synthesis and Characterization of Fe/BN Materials

**FIGURE 1:**
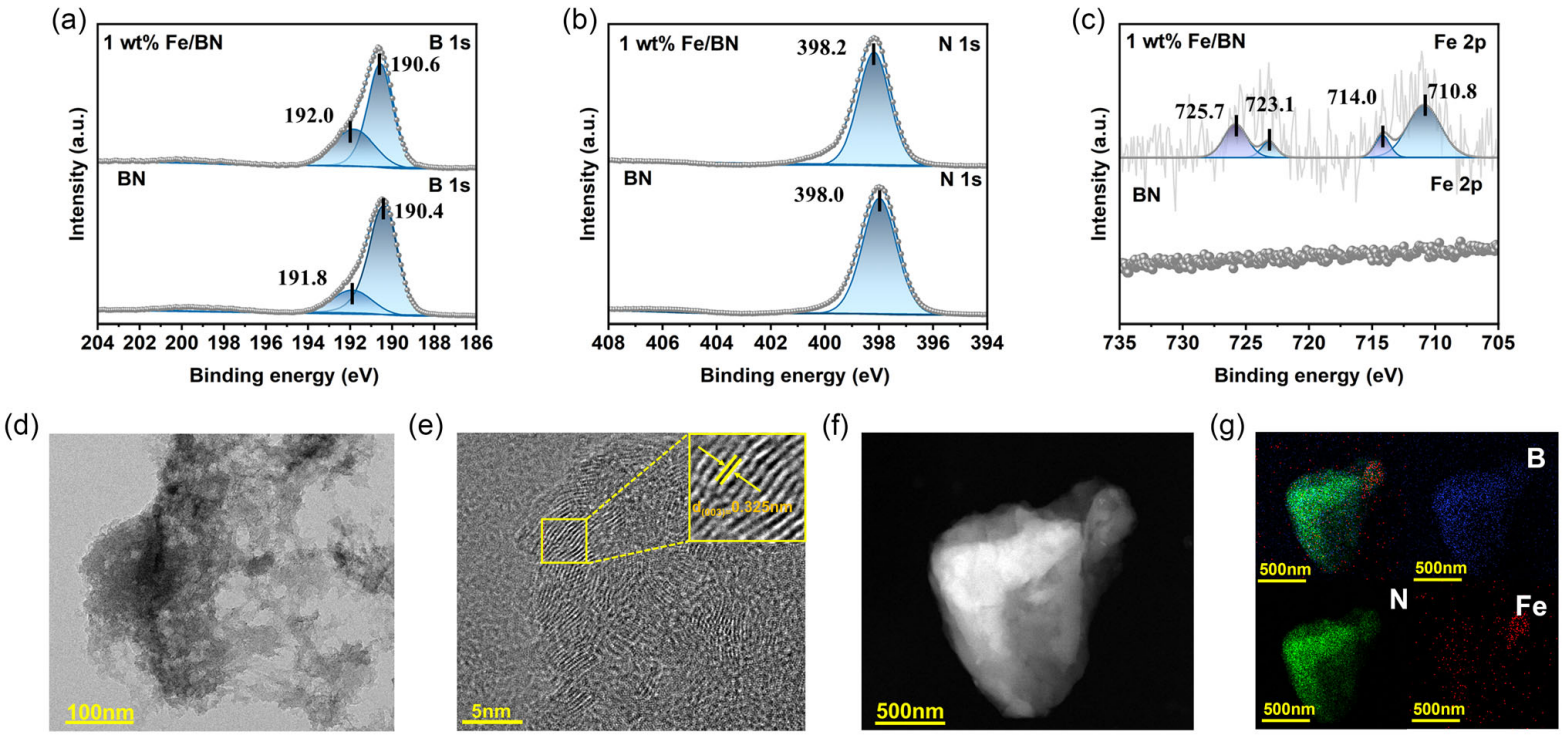
(a) B 1s, (b) N 1s, and (c) Fe 2p XPS spectra of BN and 1 wt% Fe/BN. (d) TEM and (e) HRTEM images of 1 wt% Fe/BN. (f) HAADF image and (g) the corresponding EDS elemental mapping of 1 wt% Fe/BN.

Fe/BN photocatalysts were synthesized by preparing BN nanosheets via high‐temperature calcination, followed by Fe deposition onto the BN surface using a photodeposition technique [38]. By systematically controlling the amount of Fe precursor, three samples with different loadings—0.5 wt% Fe/BN, 1 wt% Fe/BN, and 1.5 wt% Fe/BN—were prepared. X‐ray diffraction (XRD) was used to examine the crystal structures of the Fe/BN materials (Figure S1). The XRD pattern of BN agrees well with the standard PDF card (PDF#45−1171), where the peaks at 2θ = 26.7° and 42.6° correspond to the (003) and (101) planes of BN [39, 40], confirming successful BN preparation. Notably, the Fe/BN samples show no obvious peak shift relative to pristine BN, indicating that Fe introduction does not disrupt the BN lattice. The absence of Fe‐related diffraction peaks suggests that either the Fe loading is below the detection limit or the Fe species are highly dispersed without forming crystalline particles. As shown in Table S1, the actual loading of Fe in the 1 wt% Fe/BN material has been determined by inductively coupled plasma mass spectrometry (ICP‐MS) to be 0.924%. To probe surface composition, valence states, and interfacial interactions, X‐ray photoelectron spectroscopy (XPS) was performed. The high‐resolution XPS spectra and survey are shown in Figures 1 and S2, respectively. As shown in Figure 1a,b B 1s and N 1s peaks of BN appear at 190.4 and 398.0 eV, consistent with B—N covalent bonds in BN [41]. After Fe incorporation, both peaks shift by ~0.2 eV toward higher binding energies, indicating electronic interaction between Fe and BN. Meanwhile, Figure 1c shows no Fe signal for BN, whereas Fe/BN exhibits peaks at 710.8 and 723.1 eV [42], assigned to Fe 2p_3/2_ and Fe 2p_1/2_, confirming successful Fe loading on BN mainly as ionic species.

To systematically investigate the microscopic morphology of the Fe/BN photocatalytic material and the dispersion state of Fe species, transmission electron microscopy (TEM) and high‐resolution transmission electron microscopy (HRTEM) were employed for comparative characterization of both BN and Fe/BN materials. The TEM image of the pristine BN clearly shows an irregular particle morphology, with observable lattice fringes corresponding to the (003) crystal plane of BN (Figure S3). A detailed analysis of the crystal morphology of 1 wt% Fe/BN is presented in Figure 1d,e. The TEM and HRTEM characterization results of 1 wt% Fe/BN are nearly identical to those of pure BN. The dominant lattice fringes observed in the images are still attributed to the (003) plane of BN, with no characteristic particles or lattice signals corresponding to metallic Fe or Fe‐based compounds being detected [43]. This observation is consistent with the absence of Fe‐related diffraction peaks in the earlier XRD analysis, further confirming that the Fe species did not form a distinct crystalline phase. To visually confirm the presence and macroscopic distribution of Fe, this study conducted an energy‐dispersive X‐ray spectroscopy (EDS) elemental mapping analysis on the 1 wt% Fe/BN material. As shown in Figure 1f,g, the B and N elements are uniformly distributed across the examined area, and the presence of Fe species is clearly identified. Based on the comprehensive analysis results, it can be confirmed that Fe has been successfully loaded onto the surface of the BN material. The Fe species, due to their extremely small particle size and high dispersion on the BN surface under low loading conditions, did not form crystalline particles that could be directly observed.

### 2.2 Photocatalytic CH_4_ Oxidation Performance

**FIGURE 2:**
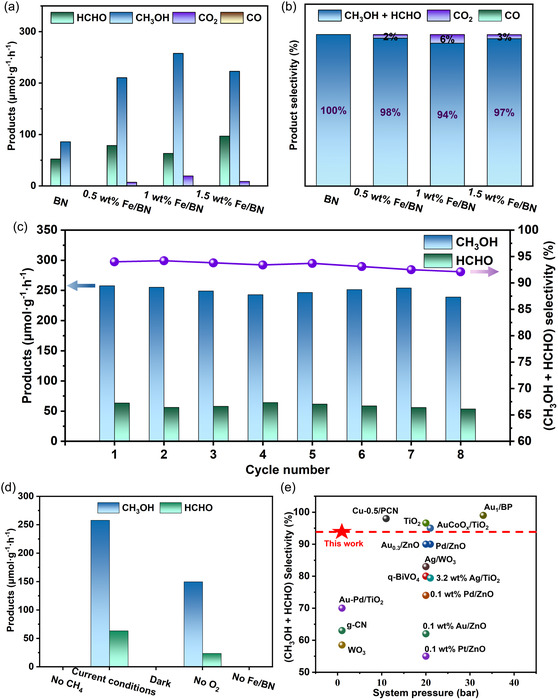
Photocatalytic methane conversion performance: (a) Comparison of production rates for different products and (b) product selectivity after 2 h of illumination. (c) Stability test of 1 wt% Fe/BN. (d) Photocatalytic CH_4_ oxidation performance under controlled single‐variable reaction conditions. (e) Comparison of (CH_3_OH and HCHO) selectivity between 1 wt% Fe/BN in a sealed system and reported photocatalysts. (Reaction conditions: 20 mg catalyst, 20 mL water, 300 W xenon lamp (full spectrum, 660 mW·cm^−2^), 60°C, ambient pressure).

To quantitatively evaluate the photocatalytic performance of the Fe/BN materials, methane conversion experiments were designed to systematically characterize the catalytic activity and product selectivity of each sample. The relevant results are presented in Figure 2 and Table S2. As shown in Figure 2a, the CH_3_OH yields of the materials exhibit a typical “volcano‐type” distribution. Compared to pure BN, all Fe‐loaded samples show significantly enhanced photocatalytic activity. Among them, the 1 wt% Fe/BN sample demonstrates the best catalytic performance, achieving an oxygenated liquid product yield of 321.1 µmol·g^−1^·h^−1^, while the yield of pure BN is only 138.3 µmol·g^−1^·h^−1^, representing a substantial increase in C1 chemical production. As the reaction time extends, the yields of all products gradually increase (Figures S4 and S5). After 2 h of reaction, the cumulative CH_3_OH yields of all Fe/BN systems exceed that of pure BN, with the 1 wt% Fe/BN sample reaching 515.5 µmol·g^−1^, further confirming its excellent catalytic activity. Figure 2b illustrates the differences in product selectivity among the various materials. Notably, BN exhibits excellent product selectivity, which aligns with previously reported results, effectively suppressing deep oxidation. The resulting products are almost exclusively CH_3_OH and HCHO, with the selectivity for liquid oxygenates approaching 100%. After Fe loading, only minor amounts of CO and CO_2_ are generated, while the selectivity for liquid oxygenates remains as high as 94.2%. This further demonstrates the significant advantage of the Fe/BN material system in catalyzing methane conversion and regulating product selectivity.

To further investigate the stability of the Fe/BN photocatalytic system for methane oxidation, cyclic stability tests were conducted. The experimental results are presented in Figure 2c and Table S3, which show that after eight consecutive cycles, the total yield of CH_3_OH and HCHO did not decrease significantly, and product selectivity remained above 90%. As shown in Figure S6, after eight reaction cycles, the XPS spectra of the catalyst have been found to be almost identical to those of the fresh catalyst and the sample after the first cycle, indicating that no significant change in the valence state of Fe has occurred during the cycling process. Meanwhile, the post‐reaction material and the reaction solution have been analyzed by ICP‐MS. As shown in Tables S4 and S5, the actual loading of Fe in the material has remained almost unchanged, and the concentration of Fe in the reaction solution has been determined to be less than 1.15 mg·L^−1^, indicating no significant metal leaching. This result has further confirmed the chemical stability of the Fe species under the reaction conditions. To rule out interference from other experimental factors, control experiments were performed under the following conditions: without CH_4_, without catalyst, without O_2_, and in the dark. As illustrated in Figure 2d and Table S6, no CH_3_OH was produced in the absence of CH_4_, catalyst, or light, confirming that CH_3_OH is generated primarily from CH_4_ in the presence of both the catalyst and light. Under O_2_‐free conditions, the yield of CH_3_OH and HCHO was only 149.4 µmol·g^−1^·h^−1^, indicating that an oxygen‐rich environment is more favorable for methane activation. Furthermore, the comparative data presented in Figure 2e and Tables S7 and S8 have demonstrated that the Fe/BN catalytic system constructed in this study has exhibited a turnover frequency (TOF) value comparable to those of existing photocatalyst systems, along with a significant advantage in the selectivity toward C1 oxygenates. Compared to most reported photocatalytic materials, this system has shown higher selectivity toward liquid oxygenated products. In summary, the Fe/BN system demonstrates outstanding photocatalytic methane conversion performance, achieving both high C1 chemical yields and favorable liquid product selectivity under mild conditions. This work provides a valuable reference for advancing selective photocatalytic methane conversion technology.

### 2.3 Mechanism Analysis

**FIGURE 3:**
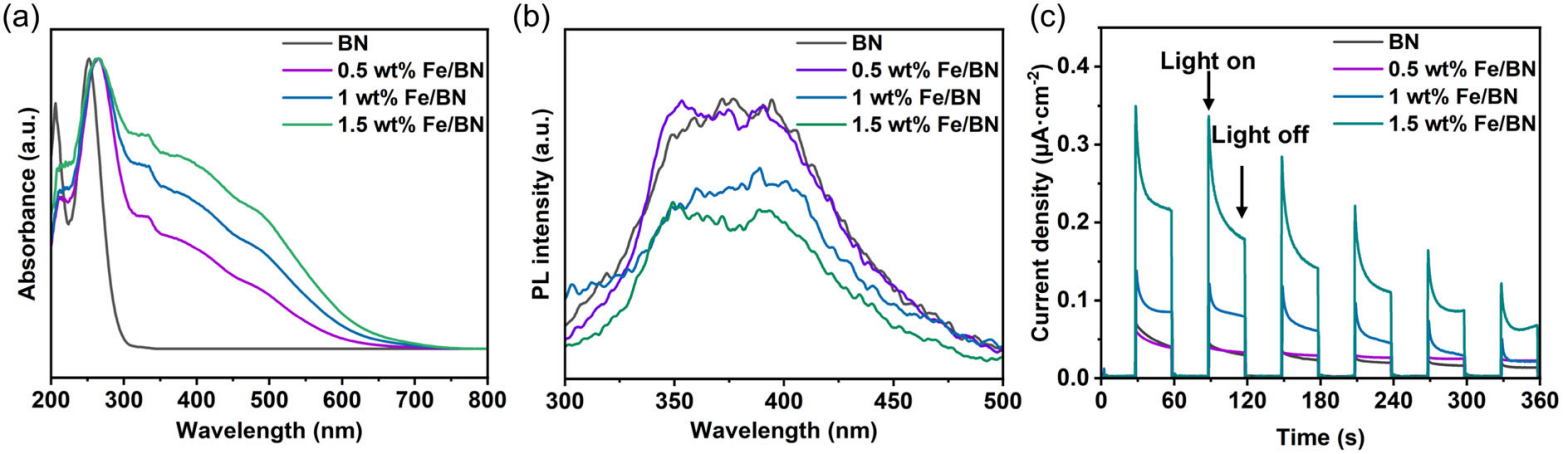
(a) UV–vis absorption spectrum of the Fe/BN composite material. (b) PL spectra. (c) Transient photocurrent response.

To elucidate the mechanism by which Fe species enhance the photocatalytic performance of BN materials in the direct conversion of CH_4_, this study systematically investigated and analyzed the light absorption properties, photogenerated carrier separation efficiency, and transient photocurrent response of the materials. The fundamental mechanism of photocatalytic reactions involves the generation of separable electron–hole pairs upon light excitation, followed by their migration to participate in surface redox reactions [44]. Collectively, the generation, migration, and surface reactions of photoinduced electron–hole pairs constitute the central processes of photocatalysis [45]. The spectral response characteristics of a photocatalyst to solar radiation serve as the primary criterion for evaluating its photocatalytic potential, as they directly determine the range and efficiency of solar energy capture. In particular, responsiveness in the visible light region is a key indicator of the practical applicability of photocatalytic materials [46]. As shown in Figure 3a, due to its wide bandgap, BN exhibits optical absorption confined strictly to the short‐wavelength ultraviolet region with negligible absorption in the visible range [47], which severely limits its practical application in photocatalytic systems [48]. In contrast, the Fe/BN composite system demonstrates significantly improved light absorption, exhibiting distinct absorption within the 400–800 nm visible region, with the absorption intensity increasing progressively with higher Fe loading [49, 50]. The band structure of the catalysts was determined based on the Kubelka–Munk‐derived bandgap (Eg) from UV–vis absorption spectra (Figure S7) and valence band XPS measurements (Figure S8) [51, 52], with a schematic representation provided in Figure S9. The results indicate that the introduction of Fe reduces the bandgap of the material, enabling it to absorb more visible light and thus generate a greater number of photogenerated carriers. In summary, Fe effectively modulates the band structure of the system and broadens its spectral response range, laying an essential structural foundation for the efficient application of this material in visible‐light‐driven photocatalytic CH_4_ conversion [53].

In photocatalytic reactions, the generation efficiency and separation capability of photogenerated charge carriers significantly influence the overall photocatalytic performance of a material. Photogenerated charge carriers must first achieve effective separation and migrate to the material's surface before they can participate efficiently in redox reactions [54, 55]. When photogenerated electrons and holes recombine, characteristic photoluminescence (PL) signals are produced. Therefore, PL spectroscopy provides a direct means to compare the photogenerated carrier separation capabilities of different samples, where PL peak intensity correlates positively with carrier recombination rates [56]. As shown in Figure 3b, pristine BN exhibits a strong PL peak, indicating a high rate of photogenerated carrier recombination. In contrast, after Fe loading, Fe/BN materials display significantly attenuated PL peaks. The underlying mechanism involves the formation of a Schottky barrier at the interface between Fe species, which serve as efficient electron acceptors, and the BN substrate [57]. This barrier suppresses the back migration of photogenerated electrons to the valence band while promoting their rapid transfer from the conduction band of BN to the Fe sites. Consequently, the probability of photogenerated carrier recombination is significantly reduced, thereby supplying a greater number of active charge carriers for subsequent catalytic reactions. To gain deeper insight into the dynamic migration behavior of these carriers, transient photocurrent response measurements were further conducted in this study. This technique directly reflects the efficiency of photogenerated carrier separation and their transport capability across the material interface under illumination [58]. Figure 3c demonstrates that the incorporation of Fe leads to a marked increase in the photocurrent response intensity of the BN material. This result corroborates the conclusions drawn from PL spectroscopy, jointly confirming that the loading of Fe species effectively optimizes the carrier separation and transport kinetics of BN materials.

**FIGURE 4:**
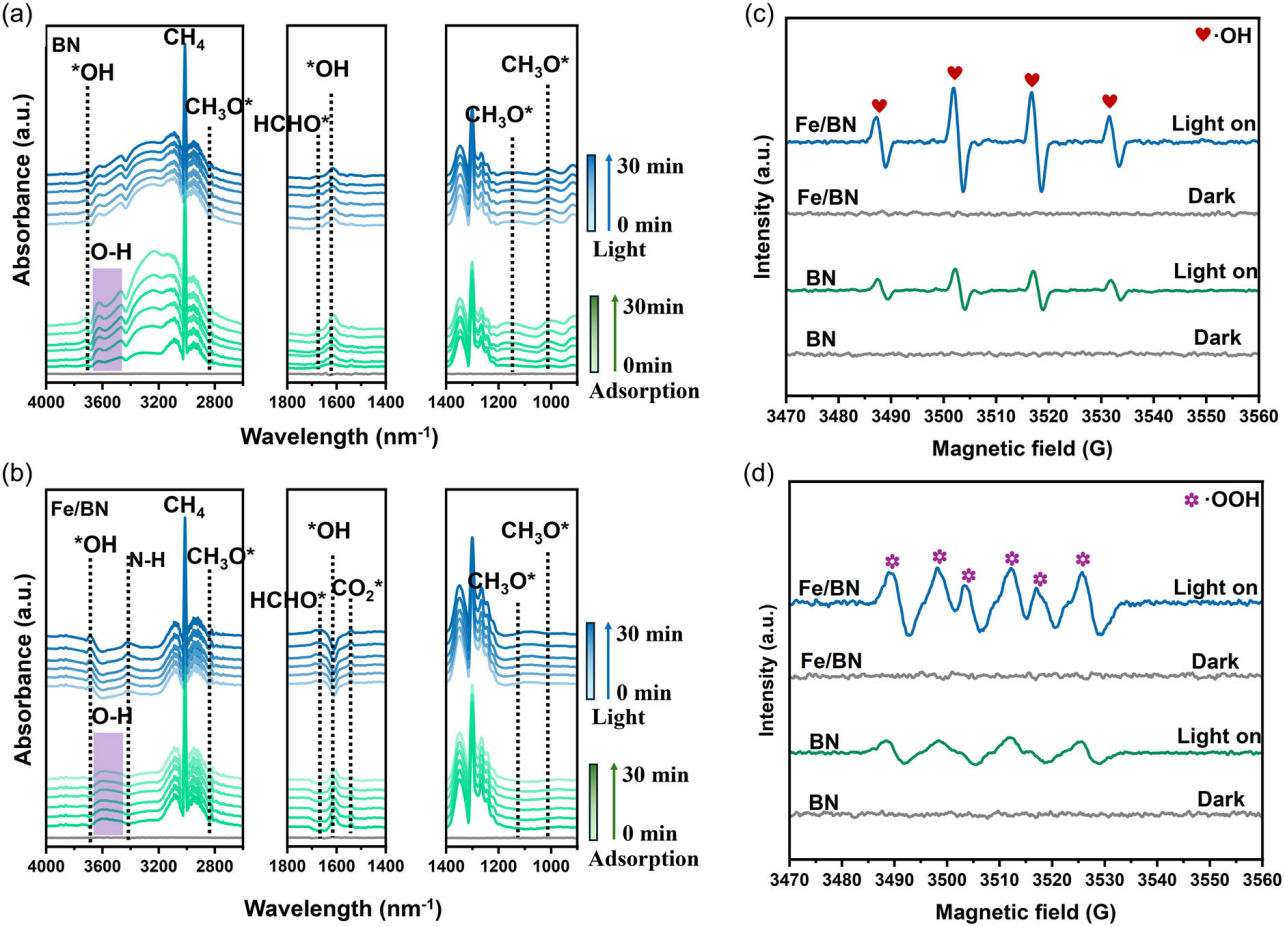
Analysis of the photocatalytic CH_4_ oxidation mechanism. (a) Enlarged in situ infrared spectra of BN and (b) Fe/BN materials. (c) In situ EPR spectra of DMPO‐·OH and (d) DMPO‐·OOH adducts.

To deeply investigate the dynamic process and reaction mechanism of photocatalytic CH_4_ conversion over Fe/BN materials and to elucidate the regulatory role of Fe species on the reaction pathways and intermediate evolution, this study selected 1 wt% Fe/BN with superior catalytic activity as the target material and employed in situ diffuse reflectance infrared Fourier transform spectroscopy for real‐time monitoring of the entire photocatalytic reaction process. By observing the emergence, intensity variation, and disappearance of characteristic infrared absorption bands during the reaction, the adsorption of reactants, formation of intermediates, and evolution of products were directly revealed, thereby clarifying the underlying reaction mechanism. During the experiment, a continuous flow of a CH_4_ and water‐vapor‐containing O_2_ mixture was introduced into the reaction system to allow sufficient adsorption of reactants onto the catalyst surface until saturation was reached. Simultaneously with the activation of the light source to start the photocatalytic reaction, the continuous collection of IR spectral data at varying illumination times was initiated. The full in situ infrared spectra of the BN and Fe/BN materials are shown in Figures 4 and S10 and S11, respectively. From the full spectra, a broad absorption band in the range of 3600–3400 cm^−1^ can be clearly observed, attributed to the asymmetric stretching vibration of O—H bonds in water molecules adsorbed on the catalyst surface [59]. Near ≈3000 and 1300 cm^−1^, signals corresponding to the asymmetric stretching and bending vibrations of C—H bonds in CH_4_ molecules were detected [52]. In the adsorption phase, the characteristic signals of H_2_O and CH_4_ intensified progressively over time and stabilized, indicating saturated adsorption of the reactants on the catalyst surface. In contrast, upon light illumination, these signals attenuated with prolonged irradiation time, confirming the continuous consumption of the reactants during photocatalysis [60].

To accurately analyze the dynamic evolution of reaction intermediates, the key spectral regions were magnified for detailed examination. The enlarged spectra for BN and Fe/BN are presented in Figure 4a,b, respectively. Under illumination, characteristic absorption peaks of hydroxyl radical species (*OH) were detected at 3684 and 1650 cm^−1^, while vibrational peaks corresponding to N—H bonds were observed around 2250 and 2450 cm^−1^ [61, 62, 63]. A characteristic absorption peak of methyl radicals (*CH_3_) was identified at 1345 cm^−1^, indicating successful activation of CH_4_. Furthermore, vibrations of the C—O bond in methoxy groups (CH_3_O*) were observed at 2897 cm^−1^ [64], and signals in the range of 1075–1100 cm^−1^ were attributed to vibrational absorption from CH_3_OH intermediates, whereas the appearance of a characteristic peak at 1586 cm^−1^ suggests the formation of adsorbed formaldehyde (HCHO*) species [65]. Based on the infrared spectroscopic evidence presented above, a reaction pathway for the photocatalytic oxidation of methane on BN materials can be reasonably proposed. The primary catalytically active site for the reaction is located at the nitrogen atom sites on the material surface [66]. Under illumination, adsorbed H_2_O molecules dissociate under the attack of photogenerated holes to form *OH radicals. The N atoms of BN nanosheets capture free protons to form N—H bonds, while CH_4_ adsorbed at the N‐atom active sites is activated by *OH to produce CH_3_O*. The CH_3_O* species then further combines with *OH to form methanol‐active species (CH_3_OH*) [67]. Compared to BN, the Fe/BN system exhibits a more pronounced *OH absorption signal at 1650 cm^−1^ and a distinct N—H vibrational peak near 3420 cm^−1^. This indicates that Fe loading may not fundamentally alter the basic reaction pathway of CH_4_ on the catalytic surface. However, Fe species serve as trapping sites for photogenerated electrons, effectively enhancing the proton capture ability of adjacent N atoms. As a result, this promotes the dissociation of H_2_O to generate more *OH and accelerates the combination of CH_4_ with *OH to form the CH_3_O* intermediate.

To further understand the role of the N—H bonds on the Fe/BN surface in suppressing overoxidation, we have analyzed the relative strength between the N—H bonds and the C—H bonds in reaction intermediates. According to literature reports [61, 62, 66], the bond dissociation energy of the C—H bond in CH_3_OH has been reported to be ≈414 kJ·mol^−1^, while those of the N—H bonds in the NH_2_ and NH species on the BN surface have been determined to be 397 and 339 kJ·mol^−1^, respectively. This has indicated that the N—H bonds are thermodynamically significantly weaker than the C—H bonds. More importantly, this study has revealed that free electrons on the BN surface tend to be injected into the antibonding orbitals of the N—H bonds, thereby inducing preferential cleavage of the N—H bonds over the C—H bonds. Based on this energetic basis, we have proposed that, in the Fe/BN system, the introduction of Fe enhances the proton‐capturing ability of the adjacent N atoms, promoting the in situ formation of N—H bonds. The formed N—H bonds, possessing lower bond dissociation energies, can preferentially break under photocatalytic conditions, releasing protons that then combine with reaction intermediates, thereby effectively suppressing the successive cleavage of C—H bonds and overoxidation. Therefore, the N—H bonds on the Fe/BN surface have served as reversible in situ proton donors, which have been identified as the key to achieving highly selective conversion of methane to C1 oxygenates.

To further elucidate the reaction mechanism of photocatalytic methane oxidation, in situ electron paramagnetic resonance (EPR) measurements were carried out using 5,5‐dimethyl‐1‐pyrroline N‐oxide (DMPO) as the spin‐trapping agent. The vacancy defects in the catalytic materials were first characterized. The results indicate that neither Fe/BN nor BN exhibits significant vacancy‐related signals (Figure S12), suggesting that the photocatalytic performance is not correlated with vacancy defects. Figure 4c presents the EPR spectrum of DMPO‐·OH adducts, where the characteristic quartet (1:2:2:1) corresponds to the formation of ·OH radicals [68]. Under illumination, both BN and Fe/BN displayed this signal, but with a marked difference in intensity. The ·OH signal for pristine BN was relatively weak, implying a limited capacity to generate ·OH. In contrast, Fe/BN showed a significantly enhanced ·OH signal, demonstrating that the introduction of Fe effectively promotes ·OH generation. Since ·OH mainly originates from water activation, this result further confirms that Fe sites enhance the ability of adjacent N atoms to capture protons, thereby accelerating H_2_O dissociation and ·OH formation. Similarly, Figure 4d presents the in situ EPR spectra of the DMPO‐·OOH adduct under light illumination. The results show that the ·OOH signal intensity generated by the Fe/BN system is significantly stronger than that of the pristine BN material. This enhancement is attributed to the high catalytic activity of the Fe sites, which effectively drives the single‐electron reduction of O_2_ to form the superoxide anion radical ·O_2_^−^ (O_2_ + e^−^ → ·O_2_^−^) [69]. During the photooxidation of methane, ·O_2_^−^ serves as a key reactive intermediate that can initiate a cascade of reactive oxygen species (ROS) and ultimately influence product selectivity. Specifically, the ·O_2_^−^ can combine with H^+^ in solution to form ·OOH. The ·OOH radical can also directly react with ·CH_3_ to generate CH_3_OH [70]. In situ EPR tests and corresponding analyses demonstrate that the introduction of Fe species effectively enhances the activation of H_2_O and O_2_ on Fe/BN, leading to a significant increase in the generation of reactive oxygen species such as ·OH. This thereby provides key oxidative species for the selective photooxidation of methane [71].

Based on the in situ IR and EPR results described above, the regulatory role of Fe species in the reaction pathway can be reasonably inferred. First, under light excitation, BN generates photogenerated carriers, and Fe species serve as electron‐trapping sites that rapidly separate these carriers. Adsorbed water molecules are activated by photogenerated holes to form ·OH, while the N atoms on BN nanosheets capture protons to form N—H bonds. Furthermore, the N—H bonds formed on the BN nanosheets can provide protons in situ, thereby further suppressing deep oxidation. These findings indicate that Fe/BN exhibits a dual synergistic effect in radical regulation: on one hand, it promotes the generation of ·OH and ·OOH, establishing a reactive environment conducive to methane activation and selective oxidation to methanol; on the other hand, the N—H bonds on the BN nanosheets supply protons in situ, effectively inhibiting overoxidation of key intermediates such as CH_3_O*. Based on the EPR evidence and reaction pathway analysis presented, the complete photocatalytic oxidation pathway of CH_4_ on the Fe/BN surface can be summarized as: CH_4_* → CH_3_* → CH_3_O* → CH_3_OH* → HCHO*.

**FIGURE 5:**
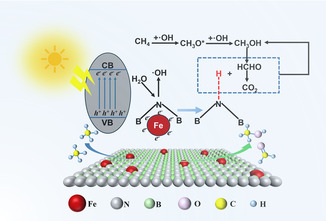
The photocatalytic methane oxidation reaction pathway over Fe/BN materials.

Based on the aforementioned experimental evidence, a reasonable reaction mechanism is proposed for the photocatalytic oxidation of methane on Fe/BN catalysts, as depicted in Figure 5. Under photoexcitation, electrons in the valence band of BN transition to the conduction band, generating photogenerated charge carriers. Acting as an electron acceptor, Fe sites facilitate the migration of photogenerated electrons, thereby significantly enhancing carrier separation efficiency. Electrons transferred to Fe sites can reduce O_2_, generating reactive oxygen species such as ·OOH. Concurrently, N atoms on the material surface capture protons from H_2_O, forming N—H bonds and promoting the generation of ·OH. CH_4_ is activated to ·CH_3_ by reactive oxygen species, and this intermediate further reacts with reactive oxygen species to undergo stepwise conversion into CH_3_OH and HCHO. It is particularly noteworthy that the N—H bonds on the material surface can provide protons in situ during the reaction, effectively suppressing overoxidation of intermediates. In summary, the introduction of Fe species effectively modulates the band structure of the Fe/BN system, markedly broadening its optical response range. Simultaneously, as an excellent electron acceptor, Fe forms a Schottky barrier with the BN matrix, thereby efficiently suppressing the recombination of photogenerated carriers. Furthermore, Fe species enhance the proton‐capture ability of adjacent N atoms, which in turn promotes the dissociation of H_2_O to generate hydroxyl radicals. This creates a richer reactive oxygen environment and an in situ proton supply for methane activation and the subsequent conversion of CH_3_O* intermediates, ultimately leading to a significant enhancement in CH_4_ conversion activity.

## 3 Conclusion

In summary, this study reports a photocatalytic material consisting of Fe supported on BN nanosheets, which enables the efficient and selective oxidation of CH_4_ to CH_3_OH and HCHO under mild conditions. After 2 h of light irradiation, the production rates of CH_3_OH and HCHO reached 321.1 µmol·g^−1^·h^−1^, representing a 2.3‐fold increase compared to pure BN material, while the selectivity for oxygen‐containing liquid compounds exceeded 94.2%. Based on in situ infrared spectroscopy and EPR characterization, the introduction of Fe species is found to modulate the band structure of BN, broadening its light‐response range. Simultaneously, Fe sites act as electron acceptors, effectively promoting charge‐carrier separation and transfer, thereby accelerating the dissociation of H_2_O to generate ·OH radicals. These reactive oxygen species subsequently activate CH_4_ molecules to form ·CH_3_ intermediates, which then combine with active oxygen species to yield CH_3_OH. Moreover, Fe sites enhance the proton capture capability of neighboring N atoms, facilitating the formation of N—H bonds. These N—H bonds can supply protons in situ, effectively suppressing the dehydrogenation of key intermediates. The N—H bonds on the Fe/BN surface have served as reversible in‐situ proton donors, which have been key to achieving the highly selective conversion of methane to C1 oxygenates. The synergistic interaction between Fe and BN transcends conventional electron transfer: Fe not only promotes photogenerated charge separation but, more importantly, alters the reaction pathway by modulating surface proton chemistry. This work is approached from the perspective of two‐dimensional materials and adopts a non‐noble metal loading strategy, providing a reference for the development and modification of new‐generation photocatalytic semiconductor materials.

## Supporting Information

Additional supporting information can be found online in the Supporting Information section.

## Funding

This study was supported by the National Science Fund for Distinguished Young Scholars (52325401), the National Natural Science Foundation of China (W2412080), the Sichuan Province Central Government‐Guided Local Science and Technology Development Special Project (2025ZYD0178), and the Sichuan Province Major Science and Technology Special Project (2023ZDZX0005).

## Conflicts of Interest

The authors declare no conflicts of interest.

## Supporting information

Supplementary Material

## Data Availability

The data that support the findings of this study are available from the corresponding author upon reasonable request.
