# Supplementary material for: Fe‐Enhanced Proton Capture on Boron Nitride Surfaces for Improved Photocatalytic Methane Conversion to C1 Chemicals
Source: Small Sci. 2026 May 11;6(5):e70302. doi: 10.1002/smsc.70302 (PMC13158702; doi:10.1002/smsc.70302)
Supplement: Supplementary file 1 — Supplementary Material [file SMSC-6-e70302-s001.pdf]

Supporting information

Fe-Enhanced Proton Capture on BN Surfaces for Improved Photocatalytic Methane Conversion to C1 Chemicals

Yong He, Wang Yu, Yuehan Cao, Sibo Wang, Kailiang Xu, Ying Zhou**

Y. He, Y. Cao Y. Zhou
State Key Laboratory of Oil and Gas Reservoir Geology and Exploitation, Southwest Petroleum University, Chengdu 610500, China Y. He, W. Yu, Y. Cao, S. Wang, K. Xu, Y. Zhou.

School of New Energy and Materials, Southwest Petroleum University, Chengdu 610500, China.
E-mail: yhcao419@163.com (Y. Cao), yzhou@swpu.edu.cn (Y. Zhou).

Part I: Experimental Section

1.1. Chemicals

All chemical reagents were used as received without further purification. These included boric acid (Cologne Chemical, analytical grade), urea (Sinopharm, analytical grade), iron (III) nitrate nonahydrate (Aladdin, analytical grade), acetylacetone (Macklin, analytical grade), and ammonium acetate (Macklin, analytical grade). Deionized water (resistivity = 18.25 $M\Omega\cdot cm^{-1}$) was used throughout the experiments.

1.2. Preparation of Catalytic Materials

Synthesis of Boron Nitride. The boron nitride (BN) used in this study was synthesized via a high-temperature calcination method. The detailed procedure is as follows: (1) 2.0 g of boric acid (H_3BO_3) and 24.0 g of urea [$CO(NH_2)_2$] were weighed into a beaker and stirred for over 30 min to obtain a homogeneous mixture. (2) The mixture was transferred into a ceramic boat, spread evenly using a spatula, and then placed slowly into a large tube furnace. The furnace tube was purged with nitrogen gas for at least 20 min to remove air. (3) Under a continuous nitrogen atmosphere, the temperature was raised to 900 °C at a heating rate of 5 $°C\cdot min^{-1}$, held at this temperature for 5 h, and then allowed to cool naturally to room temperature. The resulting sample was collected and ground into a fine powder, yielding white BN solid.

Synthesis of Fe/BN. The Fe/BN composite materials in this study were synthesized using a photodeposition method. This technique leverages the photoelectric effect of semiconductor materials to generate photoinduced electrons with reducing capabilities, which then reduce metal ions in solution to metallic particles that deposit onto the carrier surface. The specific procedure was as follows: a measured quantity of iron (III) nitrate nonahydrate and 200 mg of boron nitride were uniformly dispersed in 100 mL of deionized water. The mixture was irradiated under a light intensity of 600 $mW\cdot cm^{-2}$ for 2 hours. Subsequently, the product was collected by vacuum filtration, dried in a vacuum oven at 60 °C for 12 hours, and finally ground into a fine powder to obtain the Fe/BN samples.

1.3. Characterization of Photocatalytic Materials

The crystal structure of the samples was analyzed using X-ray diffraction (XRD, PANalytical X'Pert) under the following conditions: a scanning speed of 0.02°·s^{-1}, a scanning range of 10°–80°, a tube voltage of 40 kV, and a tube current of 40 mA, with Cu Kα radiation as the source. The elemental composition and chemical states on the surface of the catalytic materials were characterized by X-ray photoelectron spectroscopy (XPS, Thermo Scientific K-Alpha, USA). To correct for instrumental errors in binding energy data, all XPS spectra were calibrated using the standard C 1s peak at 284.80 eV, ensuring accuracy and comparability of binding energy values. The optical absorption properties of the samples were measured using a Shimadzu UV-2600 ultraviolet–visible spectrophotometer (UV–vis DRS). Measurements were performed with a scanning wavelength range of 200–800 nm and a medium scanning speed. To eliminate background interference, all diffuse reflectance spectra were referenced against high-purity $BaSO_4$ powder. The behavior of photogenerated charge carriers was analyzed using a photoluminescence spectrometer (PL, Hitachi F-700, Japan). The microscopic morphology of the samples and the distribution of active components on the support were examined by high-resolution transmission electron microscopy (TEM) equipped with an energy-dispersive X-ray spectroscopy (EDS) detector (JEOL JEM-F200, Japan).

1.4. Evaluation of Photocatalytic Activity

The photocatalytic methane conversion performance evaluation system consisted of a reaction unit and a detection unit. The reaction unit included a Perfectlight PQ256 photocatalytic reactor and a xenon lamp photocatalytic activity system. The detection unit was equipped with an Agilent gas chromatograph (GC 7890B, Agilent J&W HP-PLOT Q capillary column, FID detector) and a Shimadzu gas chromatograph (GC 2010, TDX-01 packed column, FID and TCD detectors). The experiment was conducted under ambient temperature and pressure in a sealed quartz glass reactor with high optical transmittance. The reaction temperature was maintained at 60 ± 1 °C through a cooling water bath. Quantitative analysis of gaseous products was

performed using an Agilent 7900 and a Shimadzu 2010 gas chromatograph. The detailed experimental procedure was as follows: (1) First, 20.0 mg of the photocatalytic material was uniformly dispersed in 20.0 mL of deionized water and then transferred into a 400 mL glass reactor. (2) The reactor was purged with a gas mixture of 10 % oxygen in nitrogen for 10 min, then sealed. (3) Into the sealed reactor, 5.0 mL of methane was injected. Irradiation was provided by a 300 W xenon lamp through a quartz window at a light intensity of 660 $mW{\cdot}cm^{-2}$, and the reaction temperature was maintained at 60 °C using a circulating water bath. (4) Every 0.5 h, 1.0 mL of the gas-vapor mixture from the reactor headspace was sampled and injected into the gas chromatograph. The yields of products such as CH_3OH, CO, and CO_2 were calculated based on their respective peak areas using calibrated standard curves.

The principle of formaldehyde detection by colorimetry is based on the colorimetric reaction between formaldehyde and specific chemical reagents, enabling the quantitative determination of formaldehyde content by measuring the absorbance of the colored product. In this study, the acetylacetone colorimetric method was employed to determine the formaldehyde content in the liquid-phase products of photocatalytic methane conversion. This method relies on the reaction between formaldehyde and acetylacetone, forming a yellow-colored compound, and the formaldehyde concentration is quantified by measuring the absorbance at 412 nm using a spectrophotometer. The specific operational procedure is as follows: (1) 15 g of ammonium acetate, 0.3 mL of glacial acetic acid, and 0.2 mL of acetylacetone were dissolved in deionized water and diluted to 100 mL to prepare the chromogenic reagent. (2) In a colorimetric tube, 0.5 mL of the chromogenic reagent, 2.0 mL of deionized water, and 0.5 mL of the sample solution were mixed to form a total reaction volume of 3.0 mL. (3) The mixture was incubated in a constant-temperature water bath at 80 °C for 5 min. The concentration of HCHO in the liquid product was then determined using a pre-established standard curve. (4) A standard curve of absorbance versus concentration was prepared using formaldehyde standard solutions.

The HCHO concentration in the sample was calculated based on the measured absorbance value.

1.5 Electrochemical measurements

The photoelectrochemical performance of the materials has been analyzed by means of transient photocurrent response measurements. A CHI 760E electrochemical workstation manufactured by Shanghai Chenhua Instrument Co., Ltd. has been employed for the tests, and a classical three-electrode system has been adopted to construct the reaction circuit. A sample film with a uniform thickness of 50 μm has been prepared on the surface of FTO (fluorine-doped tin oxide) conductive glass, and the film has been cut to an effective test area of 1.5 × 1.5 cm^2 to serve as the working electrode. A Pt wire electrode has been used as the counter electrode, an Ag/AgCl electrode as the reference electrode, and a bias voltage of +0.3 V has been applied. A 0.5 $mol{\cdot}L^{-1}$ Na_2SO_4 solution has been used as the electrolyte. The light source employed during the measurements has been kept consistent with that used in the photocatalytic reaction, namely a 300 W xenon lamp providing full-solar-spectrum irradiation at an intensity of 660 $mW{\cdot}cm^{-2}$.

1.6. In situ diffuse reflectance infrared Fourier transform spectroscopy (in situ DRIFTS) testing

This study employed an in situ diffuse reflectance infrared Fourier transform spectroscopy (in situ DRIFTS) system to dynamically monitor the surface reaction behavior of the catalytic materials. The system consists of a Harrick reaction cell with circulating cooling water and a Bruker infrared spectrometer equipped with a liquid nitrogen-cooled mercury cadmium telluride (MCT) detector. The sample was first pretreated by purging with argon at a flow rate of 60 $mL{\cdot}min^{-1}$ for 20 minutes at 110 °C. After cooling to room temperature, a mixture of CH_4, O_2, and water vapor was introduced for adsorption, which continued for 20 minutes until adsorption equilibrium was reached, with data collected at appropriate intervals. Following adsorption equilibrium, irradiation was carried out using a 300 W xenon lamp as the

light source, and data were collected at appropriate intervals until the acquired spectra showed no further changes, at which point the test was concluded.

Part II: Supplementary Results

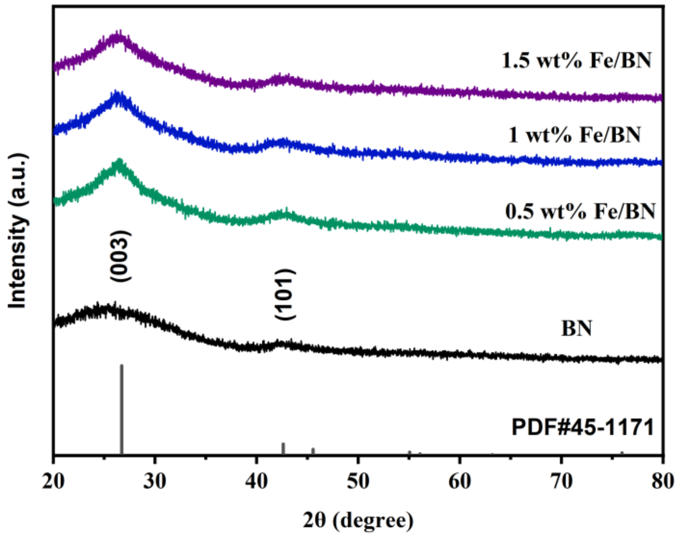

Figure S1. XRD patterns of BN and Fe/BN system materials.

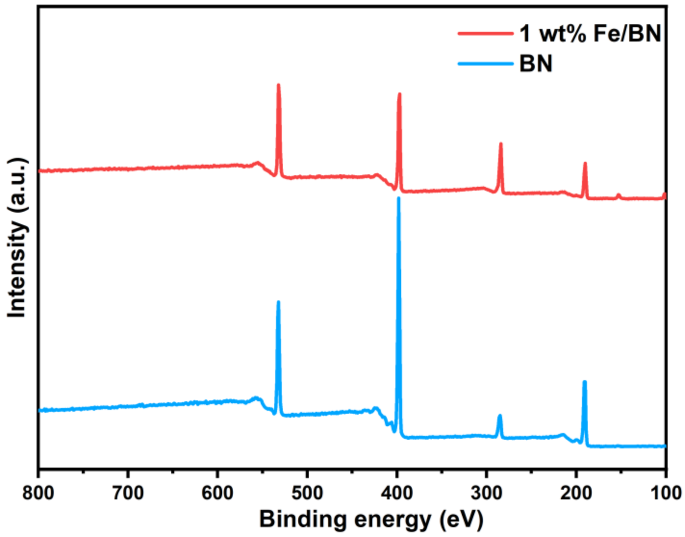

Figure S2. XPS survey spectra of BN and Fe/BN system materials.

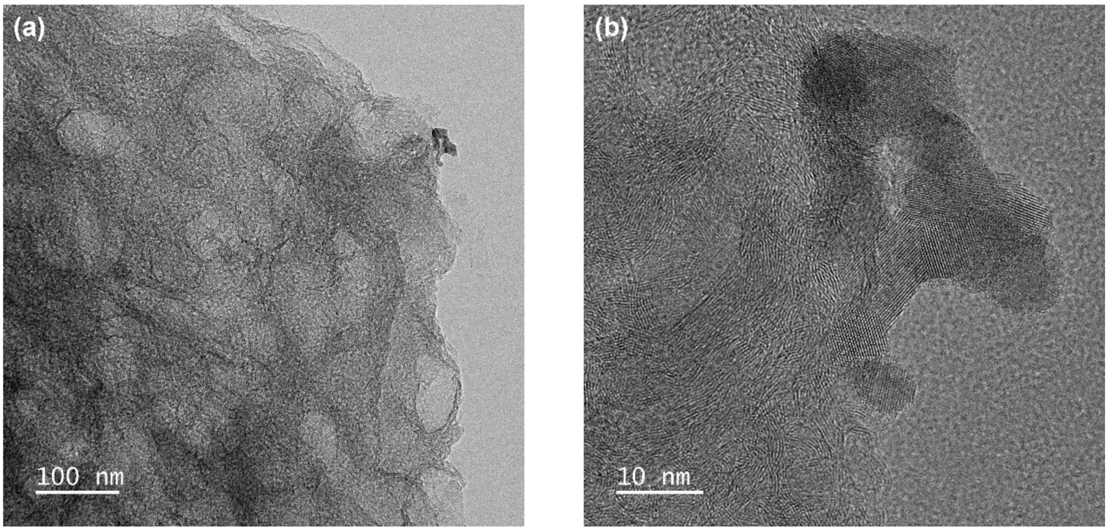

Figure S3. TEM and HRTEM images of BN.

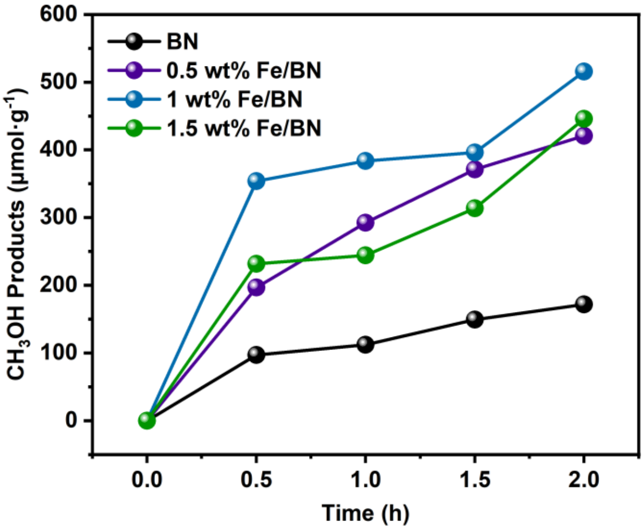

Figure S4. Time-dependent CH_3OH production profiles of BN and Fe/BN system materials.

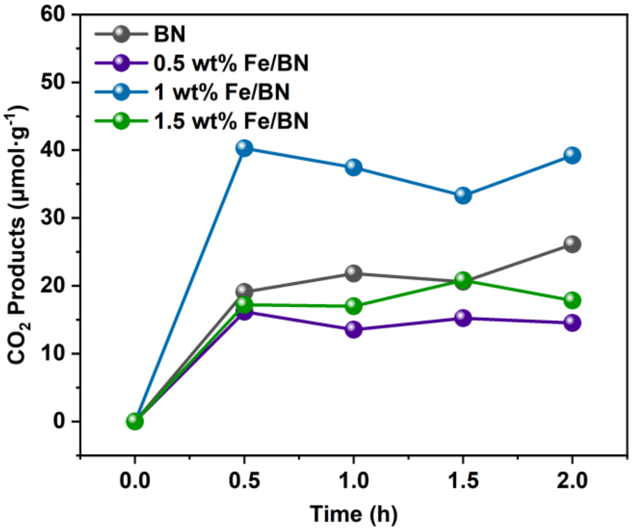

Figure S5. Time-dependent CO_2 profiles of BN and Fe/BN system materials.

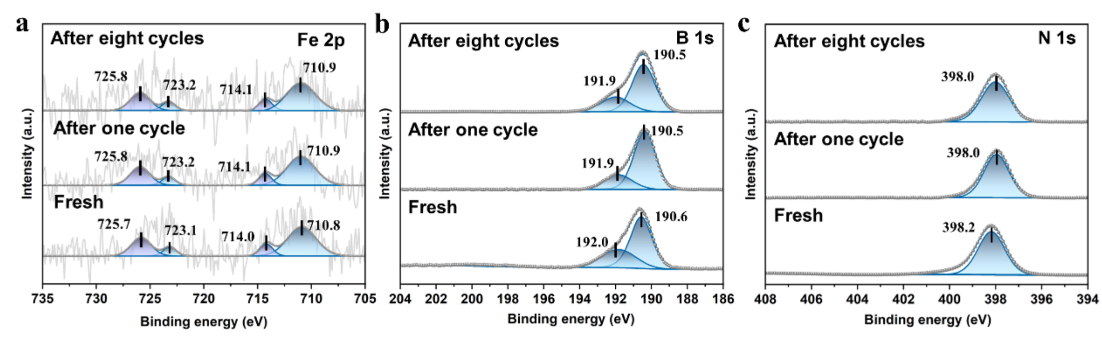

Figure S6. XPS spectra of fresh Fe/BN and those after one and eight cycles.

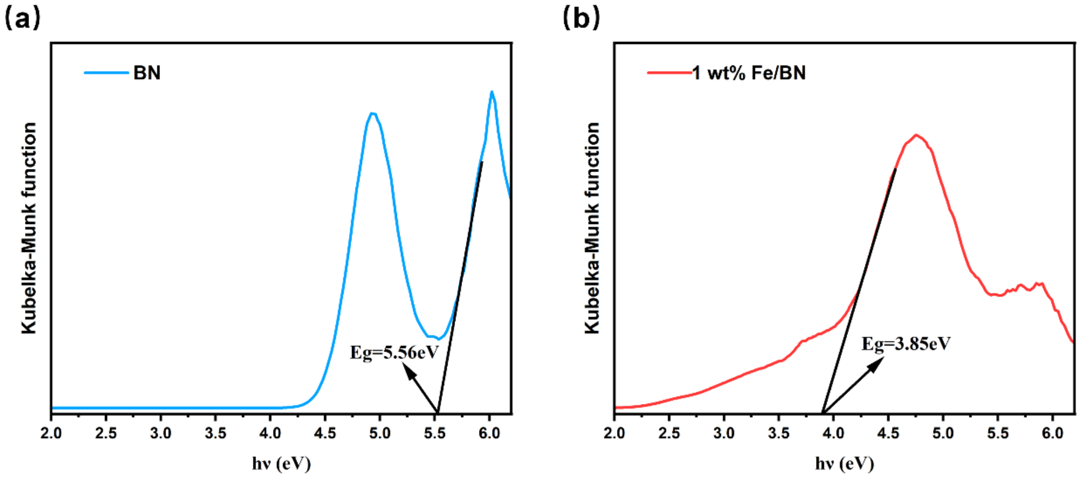

Figure S7. Kubelka-Munk plots derived from UV-Vis diffuse reflectance spectra for band gap determination of BN and Fe/BN system materials.

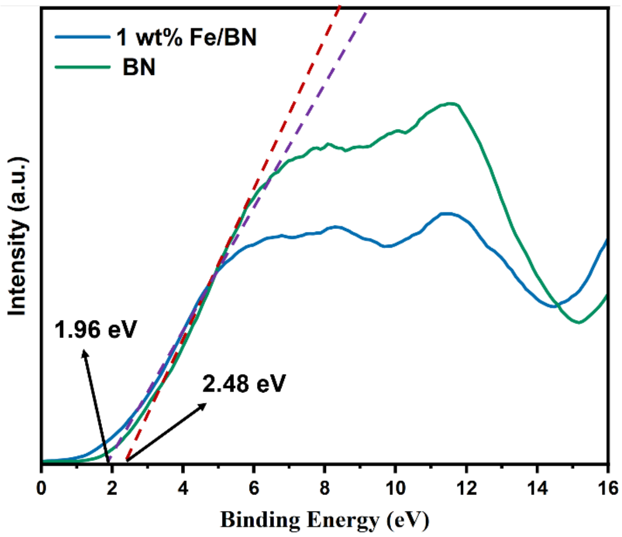

Figure S8. Valence band XPS spectra of BN and Fe/BN.

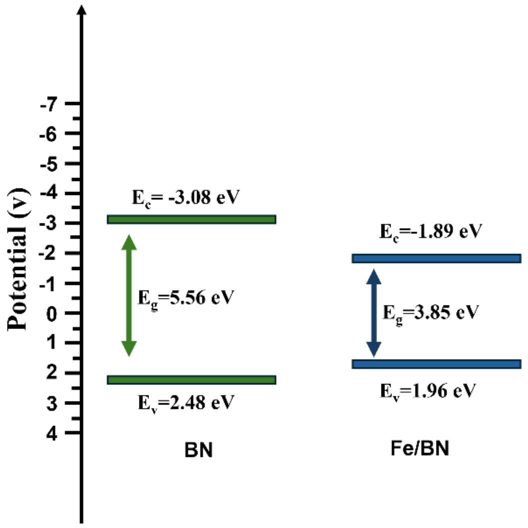

Figure S9. Band structures of BN and Fe/BN.

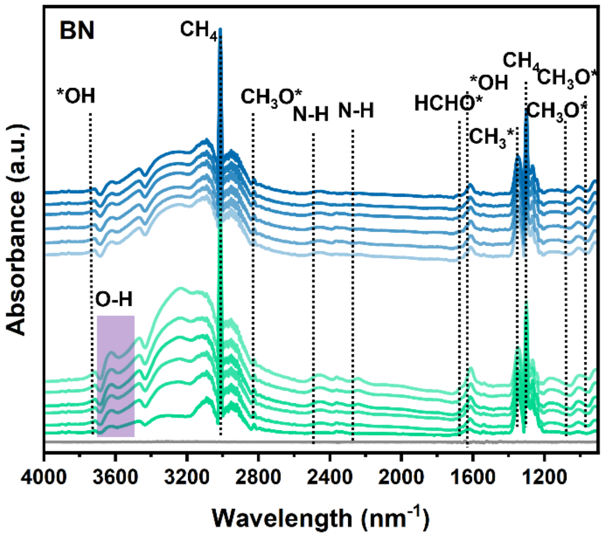

Figure S10. In situ DRIFTS spectra of BN under mixed $H_2O/CH_4/O_2$ atmosphere.

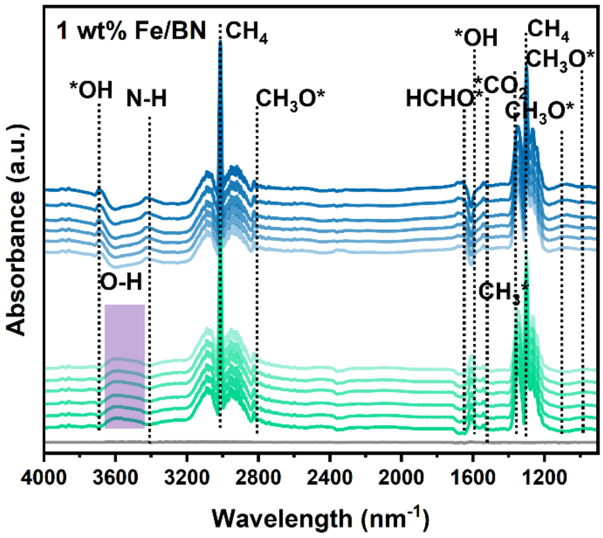

Figure S11. In situ DRIFTS spectra of Fe/BN under mixed $H_2O/CH_4/O_2$ atmosphere.

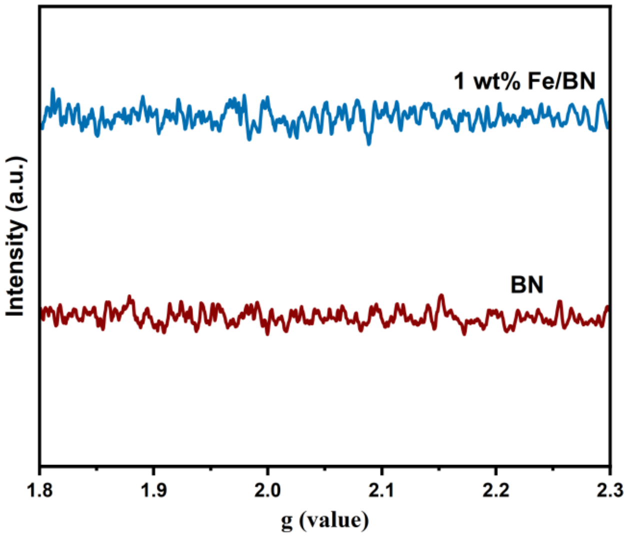

Figure S12. Vacancy defect characterization of BN and Fe/BN.

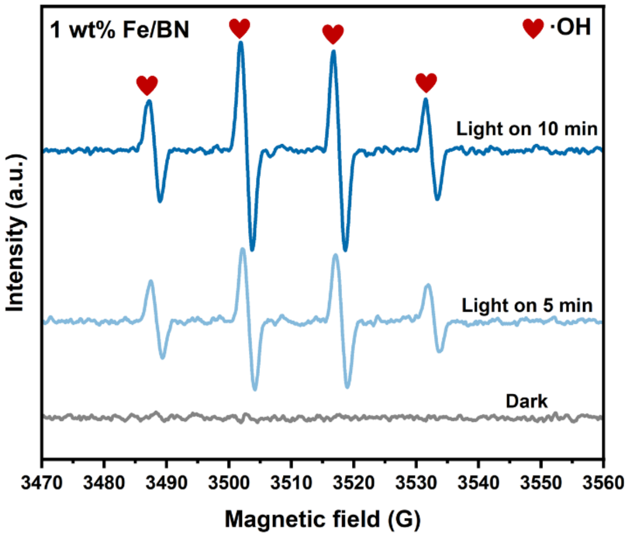

Figure S13. EPR spectra of DMPO–·OH adducts under dark conditions and different irradiation times.

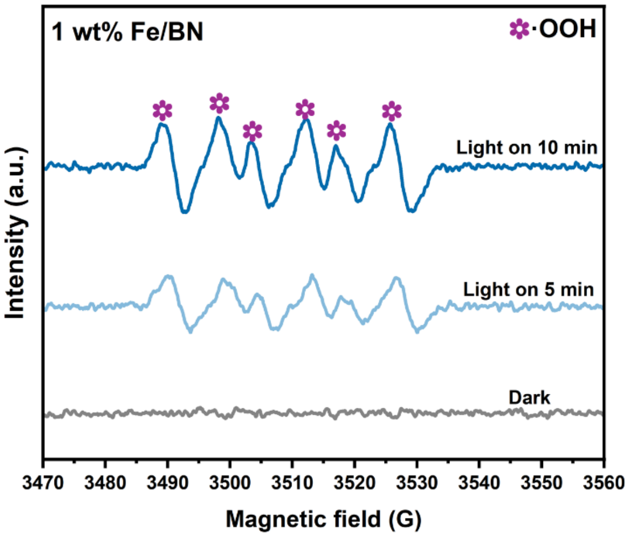

Figure S14. EPR spectra of DMPO–·OOH adducts under dark conditions and different irradiation times.

Table S1. Actual Fe loading in the Fe/BN material as determined by ICP-MS.

sample number	Fe content (wt%)	sample number	Fe content (wt%)	sample number	Fe content (wt%)	/	Fe content (wt%)
1	0.9241	2	0.9220	3	0.9255	average	0.9239

Table S2. Product formation rates and methane conversion of BN and Fe/BN system materials after 2 h of photocatalytic methane conversion reaction.

Catalyst	CH_3OH (μmol·g^{-1}·h^{-1})	HCHO (μmol·g^{-1}·h^{-1})	CO_2 (μmol·g^{-1}·h^{-1})	CO (μmol·g^{-1}·h^{-1})	Conversion rate of CH_4 (%)	Carbon balance(%)
BN	85.9	52.4	0	0	2.6	95.2
0.5 wt% Fe/BN	210.5	78.8	7.3	0.08	5.6	93.8
1 wt% Fe/BN	257.8	63.3	19.6	0	6.3	93.4
1.5 wt% Fe/BN	222.9	97.0	8.9	0.18	6.1	95.7

The calculation method is as follows:

Carbon balance (%) = $n(product)_{all}/(n(Converted\ CH_4)$

CH_4 conversion rate (%)=$(n(CH_4)_{before\ reaction}-n(CH_4)_{after\ reaction})/n(CH_4)_{before\ reactio}$

Table S3. Cycling stability test of Fe/BN.

Cycle number	CH_3OH (μmol·g^{-1}·h^{-1})	HCHO (μmol·g^{-1}·h^{-1}))	CO_2 (μmol·g^{-1}·h^{-1})	CO (μmol·g^{-1}·h^{-1})	CH_3OH+HCHO selectivity (%)
1	257.8	63.3	19.6	0	94.2
2	255.3	56.3	19.0	0.2	94.2
3	249.1	58.0	20.1	0.22	93.8
4	242.9	64.3	21.4	0.28	93.4
5	246.6	61.6	20.4	0.29	93.7
6	251.5	58.6	22.3	0.68	93.1
7	254.0	56.0	24.3	0.8	92.5
8	239.1	53.6	24.5	0.62	92.1

Table S4. Fe content in the Fe/BN material after the reaction, as determined by ICP-MS.

sample number	Fe content (wt%)	sample number	Fe content (wt%)	sample number	Fe content (wt%)	/	Fe content (wt%)
1	0.9214	2	0.9245	3	0.9234	average	0.9231

Table S5. The Fe concentration in the reaction solution as determined by ICP-MS.

sample number	Fe content ($mg{\cdot}L^{-1}$)	sample number	Fe content ($mg{\cdot}L^{-1}$)	sample number	Fe content ($mg{\cdot}L^{-1}$)	/	Fe content ($mg{\cdot}L^{-1}$)
1	1.1387	2	1.1424	3	1.1610	average	1.1474

Table S6. Product yields of Fe/BN materials under controlled single-variable reaction conditions.

Reaction Conditions	CH_3OH ($\mu mol{\cdot}g^{-1}{\cdot}h^{-1}$)	HCHO ($\mu mol{\cdot}g^{-1}{\cdot}h^{-1}$)	CO_2 ($\mu mol{\cdot}g^{-1}{\cdot}h^{-1}$)	CO ($\mu mol{\cdot}g^{-1}{\cdot}h^{-1}$)
No CH_4	0	0	0	0
No Fe/BN	0	0	0	0
No O_2 (Replaced by Ar)	149.4	23.6	6.4	0.11
Dark	0	0	0	0
Current Reaction Conditions	257.8	63.3	19.6	0

Table S7. Comparison of methane oxidation performance over photocatalytic materials.

Catalysts	Pressure (bar)	CH_3OH+HCHO selectivity (%)	Reference
Pd/ZnO	21	90	[1]
3.2 wt% Ag/TiO_2	21	79.4	[2]
TiO_2	21	96.6	[2]
0.33 metal wt% FeO_x/TiO_2	~1	90	[2]
Au_1/BP	33	99	[3]
$Au_{0.3}$/ZnO	20	90	[3]
q-$BiVO_4$	20	80	[4]
WO_3	~1	58.5	[5]
Ag/WO_3	20	83	[5]
$AuCoO_x/TiO_2$	21	95	[6]
Cu-0.5/PCN	11	98	[7]
0.1 wt% Au/ZnO	20	62	[8]
0.1 wt% Pd/ZnO	20	74	[8]
0.1 wt% Pt/ZnO	20	55	[8]
g-CN	~1	63	[9]
Au-Pd/TiO_2	~1	70	[10]
Fe/BN	~1	94.2	This work

Table S8. Comparison of TOF performance among different materials.

Catalysts	reaction conditions	Turnover Frequency (TOF, h^{-1})	Reference
0.33 metal wt% FeO_x/TiO_2	catalyst :10 mg, 25 ℃, 1 bar	6	[2]
Au_1/BP	catalyst :10 mg, 90 ℃, 33 bar	5.6	[3]
Au-Pd/TiO_2	catalyst :10 mg, 90 ℃, 70 bar	25	[10]
RhZn-MoS_2/TiO_2	catalyst :25 mg, 65 ℃, 17 bar	62	[11]
Pt/TiO_2	/	20	[12]
Cu-N_2O_1/CN	catalyst :20 mg, 25 ℃, 1 bar	13.9	[13]
Fe/BN	catalyst :20 mg, 60 ℃, 1 bar	4.12	This work

The calculation method is as follows:

$TOF=n(product)_{all}/(n_{(Fe\ sites)}\times t)$

References:

1. Yang J, Hao J, Wei J, et al. Visible-light-driven selective oxidation of methane to methanol on amorphous FeOOH coupled m-WO_3. Fuel, 2020, 266: 117104. https://doi.org/10.1016/j.fuel.2020.117104.
2. Xie J, Jin R, Li A, et al. Highly selective oxidation of methane to methanol at ambient conditions by titanium dioxide-supported iron species. Nature Catalysis, 2018, 1(11): 889-896. https://doi.org/10.1038/s41929-018-0170-x.
3. Luo L, Luo J, Li H, et al. Water enables mild oxidation of methane to methanol on gold single-atom catalysts. Nature Communications, 2021, 12(1): 1218. https://doi.org/10.1038/s41467-021-21482-z.
4. Fan Y, Zhou W, Qiu X, et al. Selective photocatalytic oxidation of methane by quantum-sized bismuth vanadate. Nature Sustainability, 2021, 4(6): 509-515. https://doi.org/10.1038/s41893-021-00682-x.
5. Zeng Y, Tang Z, Wu X, et al. Photocatalytic oxidation of methane to methanol by tungsten trioxide-supported atomic gold at room temperature. Applied Catalysis B: Environmental, 2022, 306: 120919. https://doi.org/10.1016/j.apcatb.2021.120919.
6. Song H, Meng X, Wang S, et al. Selective Photo-oxidation of Methane to Methanol with Oxygen over Dual-Cocatalyst-Modified Titanium Dioxide. ACS Catalysis, 2020, 10(23): 14318-14326. https://doi.org/10.1021/acscatal.0c04329.
7. Zhou Y, Zhang L.Wang W. Direct functionalization of methane into ethanol over copper modified polymeric carbon nitride via photocatalysis. Nature Communications, 2019, 10(1): 506. https://doi.org/10.1038/s41467-019-08454-0.
8. Song H, Meng X, Wang S, et al. Direct and Selective Photocatalytic Oxidation of CH_4 to Oxygenates with O_2 on Cocatalysts/ZnO at Room Temperature in Water. Journal of the American Chemical Society, 2019, 141(51): 20507-20515. https://doi.org/10.1021/jacs.9b11440.
9. Xie P, Ding J, Yao Z, et al. Oxo dicopper anchored on carbon nitride for selective oxidation of methane. Nature Communications, 2022, 13(1): 1375. https://doi.org/10.1038/s41467-022-28987-1.
10. AbRahim M H, Forde M M, Jenkins R L, et al. Oxidation of Methane to Methanol with Hydrogen Peroxide Using Supported Gold–Palladium Alloy Nanoparticles. Angewandte Chemie International Edition, 2013, 52(4): 1280-1284. https://doi.org/10.1002/anie.201207717.
11. Li Y, Liu H, Mao J, et al. MoS_2-confined Rh-Zn atomic pair boosts photo-driven methane carbonylation to acetic acid. Nature Communications, 2025, 16(1): 487. 10.1038/s41467-024-54061-z.
12. Zhang P, Li J, Huang H, et al. Platinum Single-Atom Nests Boost Solar-Driven Photocatalytic Non-Oxidative Coupling of Methane to Ethane. Journal of the American Chemical Society, 2024, 146(34): 24150-24157. 10.1021/jacs.4c08901.
13. Zhang Y, Guan Z, Zhang C, et al. Reactive Oxygen Species-Independent Light-Driven Selective Methane Upgrading to Ethanol over Single Cu-N_2O_1 Sites Anchored on Carbon Nitride. CCS Chemistry, 0(0): 1-15. doi:10.31635/ccschem.025.202506415.
